# A precise splice: proper splicing patterns required for appropriate lipid metabolism during callus induction with cytokinin

**DOI:** 10.1093/plphys/kiag270

**Published:** 2026-05-06

**Authors:** Alyssa Kearly

**Affiliations:** Assistant Features Editor, Plant Physiology, American Society of Plant Biologists, Rockville, MD, United States; Boyce Thompson Institute, Cornell University, Ithaca, NY, United States

Plant cells are highly developmentally plastic, a trait that allows them to regenerate plant tissues and organs. In nature, this plasticity is useful in restoring tissues after wounding, but it has also been harnessed in research for purposes of plant tissue culture and transgenics. A critical step in plant regeneration is the formation of callus, in which cells dedifferentiate from their specialized cell states into a pluripotent state, then proliferate to create a mass of cells that can re-differentiate. Organogenesis can then occur.

Callus formation is driven by intricate interactions between phytohormone signaling pathways. In tissue culture, a balance of auxin and cytokinin in callus-inducing media (CIM) promotes cellular dedifferentiation and proliferation, partly through the activation of transcription factors and cell cycle genes (Riou-Khamlichi et al. 1999; Dewitte et al. 2007; Atta et al. 2009; Sugimoto et al. 2010; Fan et al. 2012; Meng et al. 2017; Zhang et al. 2017). Genetics studies have also implicated very long chain fatty acids (VLCFAs) and splicing machinery in the regulation of callus formation (Shang et al. 2016; Li et al. 2025). Although both lipid homeostasis and splicing have been connected to phytohormone signaling in various biological contexts (Nobusawa et al. 2013; Takayanagi et al. 2022; Babić et al. 2025; Zhou et al. 2025), it remains unknown if/how these processes interact to mediate callus formation. In work recently published in *Plant Physiology*, Takeuchi et al. (2026) expand on their previous efforts exploring the genetic and molecular underpinnings of callus formation and uncover a link between alternative splicing and lipid signaling (Fig. 1).

**Figure 1:**
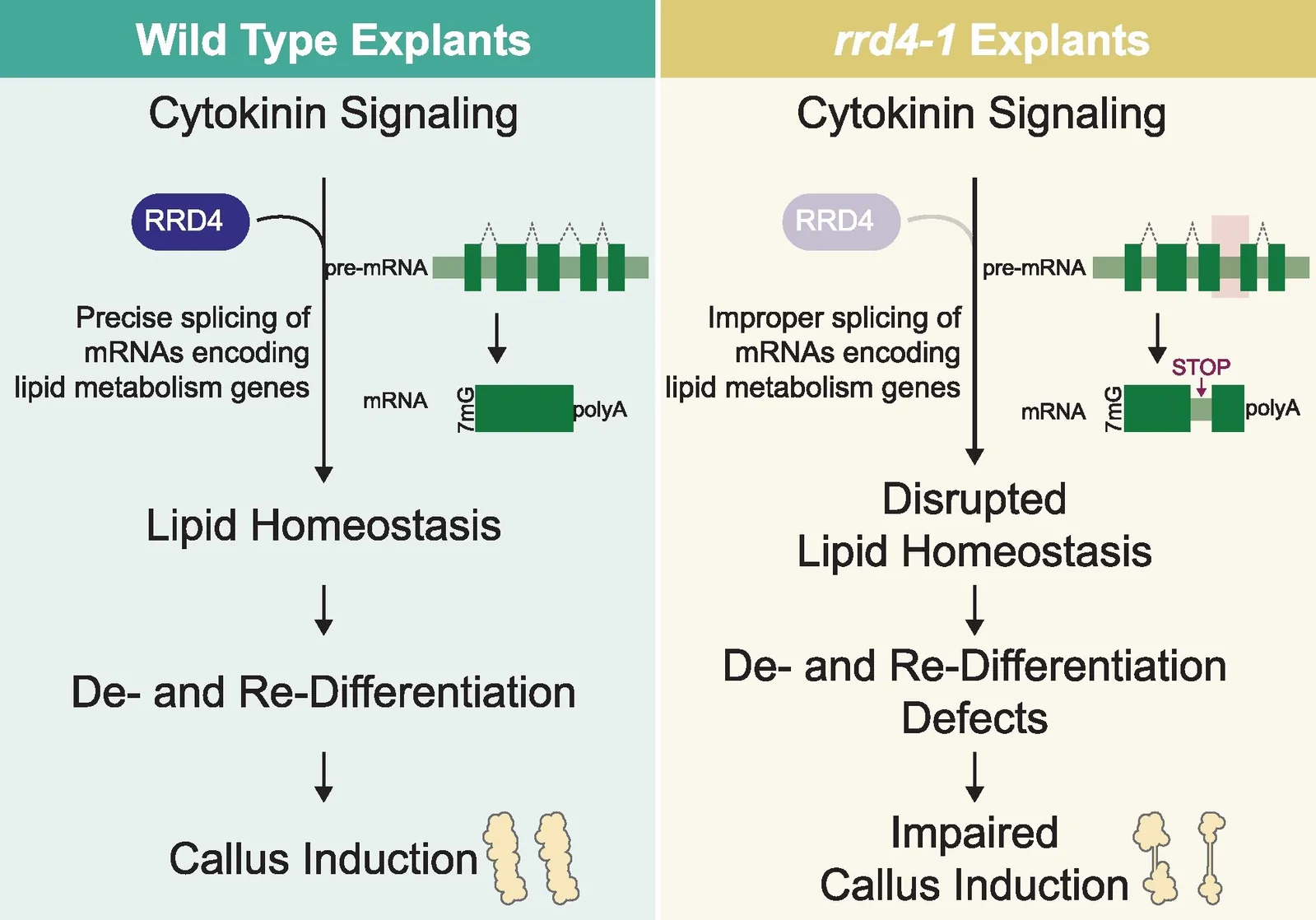
Splicing factor RRD4 impacts callus formation through regulation of alternative splicing. During callus induction, precise maintenance of splicing patterns is critical for the response to cytokinin. In *rrd4-1* mutant explants, splicing is disrupted due to the loss of splicing factor RRD4, resulting in alternative splicing events in lipid metabolism genes, presumably altering their protein expression level or function. This leads to perturbations in lipid homeostasis that impact the ability of the explants to induce callus. As heat tolerance also requires precise splicing patterns, even mild heat stress in *rrd4-1* exacerbates the callus induction phenotype.

A previous genetic screen identified mutant Arabidopsis lines with temperature-sensitive defects in plant regeneration (Sugiyama 2003). Explants from one such line, *root redifferentiation defective4-1* (*rrd4-1*), generated callus similar to wild type (WT) explants when cultured on CIM at 22°C, but were impaired at the restrictive 28°C. These findings, replicated in the current study, suggested that RRD4 plays a role in plant regeneration. However, the gene corresponding to RRD4 remained unidentified. Using chromosome mapping analysis and sequencing, Takeuchi et al. (2026) determined that the AT1G17070 gene in *rrd4-1* harbors a nonsense mutation in its open reading frame that results in a truncated form of the encoded protein. This protein is a highly conserved spliceosome disassembly factor required for the proper regulation of mRNA splicing relating to several biological processes (Tsai et al. 2005; Jones et al. 2012; Dolata et al. 2015). Notably, AT1G17070 has a documented role in heat stress-responsive alternative splicing (He et al. 2023), which could contribute to the temperature dependence of the *rrd4-1* callus induction defect. Alternative splicing may, therefore, be a key regulatory mechanism in plant regeneration.

Although *rrd4-1* explants could form callus, they had impaired root development when cultured on root-inducing media lacking cytokinin (Sugiyama 2003), suggesting a connection between cytokinin and RRD4 in regeneration that Takeuchi et al. explored further. They found that, compared to WT, cytokinin suppressed callus formation at lower concentrations in *rrd4-1* hypocotyl explants, an effect that was exacerbated at 28°C. At the molecular level, cytokinin did not upregulate RRD4 in WT or *rrd4-1* explant, but these genotypes displayed distinct splicing patterns of stress-responsive mRNAs. These findings suggest that *rrd4-1* cytokinin sensitivity is not a direct effect of RRD4 loss but may instead result from dysregulated alternative splicing of other important genes.

To dive deeper into the consequences of splicing defects on tissue regeneration, Takeuchi et al. assessed gene expression dynamics in WT and *rrd4-1* explants cultured on CIM with (CIM+) and without (CIM−) kinetin, a form of cytokinin. Within 12 hours of culture, over 1000 genes were differentially expressed between WT explants on CIM+ and CIM−, but this kinetin response was dramatically dampened in *rrd4-1* explants. Comparisons between WT and *rrd4-1* revealed distinct transcriptomic responses to CIM under both culture conditions. Interestingly, a suite of cell cycle-associated genes in the *rrd4-1* mutant were downregulated early in both CIM culture conditions, potentially contributing to their defect in callus induction. However, no broad genotype- or condition-related trend in phytohormone response-associated genes could be discerned. At the transcript expression level, therefore, it appeared that phytohormone responses were not impacted by RRD4 loss.

The molecular mechanism underlying the *rrd4-1* phenotype remained elusive after expression analyses, but given the function of RRD4 in alternative splicing, Takeuchi et al. sought to explore alternative splicing dynamics. Analyses of splicing events after 12 hours of culture revealed that loss of RRD4 greatly disrupted global splicing patterns, resulting in increased alternative splicing events regardless of kinetin presence or absence. The inclusion of kinetin in the CIM resulted in modest alterations to splicing patterns in WT explants but dramatically shifted the splicing patterns in *rrd4-1* explants, suggesting that RRD4 is required for maintaining proper splicing patterns in response to cytokinin signaling. Generally, the correlation between differential expression and differential splicing of a given transcript was minimal, suggesting that the effects of the alternative splicing events may instead be seen at the protein level, impacting the translation efficiency of transcripts or the stability or function of the encoded proteins.

The alternative splicing analyses uncovered sphingolipid biosynthesis as a process potentially impacted by kinetin and the loss of RRD4, as associated genes were enriched in key differentially spliced gene sets. Indeed, specific lipid biosynthesis-associated mRNAs had higher rates of intron retention in the *rrd4-1* explants on both CIM− and CIM+. Although the intron retention had minimal impact on expression, the retained introns were presumed to introduce premature stop codons, suggesting potential impacts on protein function or expression. Through lipidomics, lipid homeostasis was found to be perturbed in *rrd4-1* mutants, with effects further exacerbated by the presence of kinetin in the CIM. Inhibitor studies targeting fatty acid biosynthesis further supported a connection between cytokinin, RRD4, and lipid homeostasis in callus formation, linking the dysregulation of lipid biosynthesis-associated mRNA splicing to the *rrd4-1* phenotype.

The process of plant regeneration is multifaceted and intricate, underpinned by interactions between complex phytohormone pathways and external stimuli. In their characterization of the *rrd4-1* mutant, Takeuchi et al. uncovered that alternative splicing is an influential process in callus formation, particularly necessary for fine-tuning lipid metabolism in response to cytokinin. Transcriptomic analyses revealed other pathways impacted by alternative splicing, including chromatin remodeling, that provide additional directions for future research and highlight the complexity of callus induction as a biological process. Furthermore, it remains unknown how heat stress compounds with cytokinin responses to impact splicing and callus induction. Overall, plant cells have complex methods for regulating cellular de- and re-differentiation, and this work has provided insight into a mechanism by which cells integrate phytohormone signaling, alternative splicing, and lipid metabolism to coordinate these processes.

**Recent related articles in *plant physiology***

- Durán-Medina et al. (2025) describe how transcription factor ENHANCER OF SHOOT REGENERATION 2 (ESR2) regulates expression of cytokinin-associated genes to fine-tune cytokinin biosynthesis and signaling during green callus formation.
- Wang et al. (2025) identify auxin transporters in rice that, following physical damage to the root, mediate shoot-to-root auxin transport to promote compensatory lateral root growth.

## Data Availability

No new data was generated for this publication.
